# Prognostic value of atherogenic index of plasma in pulmonary hypertension

**DOI:** 10.3389/fmed.2024.1490695

**Published:** 2025-01-13

**Authors:** Meng-Qi Chen, An Wang, Chuan-Xue Wan, Bin-Qian Ruan, Jun Tong, Jie-Yan Shen

**Affiliations:** Department of Cardiology, Renji Hospital, Shanghai Jiao Tong University School of Medicine, Shanghai, China

**Keywords:** pulmonary hypertension, atherogenic index of plasma, atherosclerosis, prognosis, triglycerides, high-density lipoprotein cholesterol

## Abstract

**Background:**

The atherogenic index of plasma (AIP) is a brand-new lipid parameter that has been used to assess various cardiovascular events. This study aimed to investigate the prognostic value of AIP in patients with pulmonary hypertension (PH).

**Methods:**

This retrospective study was conducted at Shanghai Jiao Tong University School of Medicine affiliated Renji Hospital, and included data from 125 PH patients treated during 2014–2018. The endpoint events of this study were clinical worsening outcomes. PH patients include those from group 1 and group 4. AIP was determined as the logarithm of the blood triglycerides ratio to high-density lipoprotein cholesterol.

**Results:**

The 1-year, 3-year, and 5-year incidence rates of clinical worsening outcomes in PH patients in this study were 20.0, 44.8, and 54.4%, respectively. The median age of the PH patients was 38.00 years, with females accounting for 90.4%. After controlling for multivariable factors, the results of Cox regression analysis indicated that AIP was an independent predictor of adverse outcomes with a hazard ratio and 95% confident interval (CI) of 2.426 (1.021–5.763). The positive linear relationship of AIP was evaluated using restricted cubic spline analysis. Kaplan–Meier curves showed a significantly higher events rate in patients with AIP ≥ 0.144 compared to those with AIP < 0.144 (*p* = 0.002). Four potential prognostic variables, including AIP, were identified by LASSO regression to construct a nomogram. Compared to the model minus AIP, the AUC of the nomogram displayed a non-significant improvement (0.749 vs. 0.788, *p* = 0.298). In contrast, the results of net reclassification improvement (0.306, 95% CI: 0.039–0.459, *p* < 0.001) and integrated discrimination improvement (0.049, 95% CI: 0.006–0.097, *p* = 0.020) demonstrated significant enhancements in the predictive ability of the model when AIP was added to the clinical model.

**Conclusion:**

AIP is an independent predictor of long-term clinical worsening in PH patients, and its inclusion in prognostic models could improve risk stratification and management.

## Introduction

Pulmonary hypertension (PH) is one of the serious cardiovascular diseases characterized by pulmonary vascular remodeling and increased pulmonary vascular resistance (PVR), leading to heart failure and ultimately death (1). The number and burden of PH patients have been growing, with the prevalence rates of 1% globally and 10% among people over 65 years of age (2). The use of targeted therapy for PH has significantly improved clinical symptoms and quality of life, leading to a substantial increase in survival rates for PH patients. Despite advancements in treatment, the prognosis for PH remains poor, with a median survival of approximately 3–5 years from diagnosis (3). Identifying reliable prognostic markers is crucial for improving risk stratification and guiding therapeutic decisions in PH patients.

The atherogenic index of plasma (AIP) is a well-recognized and easily-measured biomarker of atherosclerosis risk that has been used to assess the risk of various cardiovascular events. AIP is a brand-new lipid parameter proposed by Dobiásová and Frohlich (4), calculated as the logarithm of the triglycerides (TG) /high-density lipoprotein cholesterol (HDL-C). It has been established that AIP may reflect the balance between atherogenic lipoproteins (such as TG-rich lipoproteins) and anti-atherogenic lipoproteins (such as HDL-C) (5, 6). In addition, studies have shown a close correlation between inflammatory cytokines and increased TG and decreased HDL-C levels in patients with idiopathic pulmonary arterial hypertension (IPAH), suggesting that AIP may serve as a novel indicator of systemic inflammation (7). A higher AIP value suggests a disturbed lipid metabolism and an increased likelihood of developing cardiovascular complications (8, 9). Numerous studies have established that AIP is a predictive marker for the risk stratification of coronary artery disease, diabetes mellitus, metabolic syndrome, and other cardiovascular conditions (10–14).

Although previous studies have explored the correlation between AIP and cardiovascular diseases, systematic research on the relationship between AIP and PH is relatively lacking. The ratio of TG to HDL-C has been reported to be associated with the risk and prognosis of IPAH (7, 15). However, the prognostic role of AIP in PH has not been sufficiently explored. Accordingly, this study aimed to investigate the prognostic value of AIP in PH patients and build a simple and reliable nomogram for early risk stratification and optimal management strategies.

## Methods

### Study population

The present study is a retrospective study based on the data of 125 patients with pulmonary hypertension who were treated at Shanghai Jiao Tong University School of Medicine affiliated Renji Hospital, between January 2014 and December 2018. 125 PH patients included 114 patients with pulmonary arterial hypertension (PAH, group 1 PH) and 11 patients with chronic thromboembolic PH (CTEPH, group 4 PH). The inclusion criteria for this study were: (1) participants aged 18 years or older; (2) patients were newly diagnosed with PH confirmed by right heart catheterization (mean pulmonary artery pressure ≥ 25 mmHg); (3) availability of relevant clinical data within 1 month before and after right heart catheterization. The exclusion criteria were: (1) participants with group 2, group 3 and group 5 PH; (2) participants with a history of malignancy, lung transplantation, and severe heart failure; (3) participants who were pregnant during the study period; (4) participants with a history of diabetes or inflammatory infection. Informed consent was obtained from all patients included in this study. This study has been approved by the Ethics Committee of Renji Hospital, Shanghai Jiao Tong University School of Medicine, Shanghai, China.

### Data collection

This study collected relevant data including patients’ demographics, World Health Organization functional class (WHO-FC), 6-min walk distance (6MWD), PH initial therapy, laboratory tests, right heart catheterization data, and echocardiography. Demographic information included age, gender, height, and weight. Laboratory tests included total cholesterol (TC), TG, HDL-C, low-density lipoprotein cholesterol (LDL-C), C-reactive protein (CRP), white blood cell (WBC), and B-type natriuretic peptide (BNP) data. Data of laboratory tests were uniformly assessed before the patients underwent right heart catheterization, at which point their clinical status was relatively stable. Echocardiography mainly collected data on right ventricular internal diameter, estimated pulmonary artery systolic pressure (PASP), and tricuspid annular systolic excursion (TAPSE). Right heart catheterization data included mean pulmonary artery pressure, right atrial pressure (RAP), pulmonary artery wedge pressure (PAWP), cardiac index, cardiac output (CO), mixed venous oxygen saturation (SvO_2_), and pulmonary vascular resistance (PVR). The clinical status of PH patients was determined through electronic medical record query or telephone follow-up.

### Definition

The primary endpoint of this study was the occurrence of clinical worsening outcome, including lung transplantation, rehospitalization, all-cause death. Follow-up time was determined as the time from right heart catheterization to the occurrence of clinical worsening outcomes. Atherogenic index of plasma was defined as the logarithm value of the ratio of triglycerides to high-density lipoprotein cholesterol (log[TG/HDL-C]).

### Statistical analysis

Continuous variables were presented as median (interquartile range). Categorical variables were presented as frequency (percentage). Comparisons between two groups were conducted using Student’s *t*-test, Mann–Whitney test, Chi-square test, and rank-sum test, depending on the type of data. Multiple imputation was performed to supplement missing variables in this study. Restricted cubic spline and Cox regression analyses were used to assess the correlation between AIP and the risk of clinical worsening events in PH patients. The optimal *p*-value method was used to determine the best cutoff value of AIP by identifying the point that maximizes the statistical significance of its association with the risk of clinical worsening in PH patients. Kaplan–Meier survival curves were used to compare the difference in survival status between high and low AIP groups in PH patients. Least absolute shrinkage and selection operator (LASSO) regression analysis was used to select potential variables related to PH prognosis. A nomogram was constructed to build a prediction model. Receiver operating characteristic (ROC) curve, net reclassification improvement (NRI), and integrated discrimination improvement (IDI) were used to evaluate the reliability of the model in predicting clinical worsening events in PH patients. All data analyses in this study were conducted using R 4.41 software and SPSS 27.0 software (IBM Corp). A *p*-value <0.05 was regarded statistically significant.

## Results

### Baseline characteristics of PH patients

The baseline characteristics of PH patients in this study are shown in Table 1. A total of 125 PH patients aged ≥18 years were included in this study. The median age of the 125 PH patients was 38.00 (30.00–51.00) years, with females accounting for 90.4% of the population. The median BMI was 21.50 (19.56–23.34) kg/m^2^, and the median 6MWD was 389.23 (311.60–465.80) meters. IPAH accounted for 21.6% of cases, connective tissue disease-associated PAH accounted for 52.0%, congenital heart disease-associated PAH accounted for 13.6%, portopulmonary hypertension (PoPH) accounted for 4.0%, and CTEPH accounted for 8.8%. The proportion of patients in WHO-FC I/II was 68.0%, while the proportion in class III/IV was 32.0%. The median BNP level was 200.00 (80.00–455.00) pg./mL. Among the clinical variables, there were statistically significant differences in 6MWD, WHO-FC, TC, LDL-C, CRP, and AIP between the two groups of PH patients with and without clinical worsening outcomes (other variables showed no statistically significant differences).

**Table 1:** Baseline characteristics of patients with pulmonary hypertension.

Characteristics	Total (125)	Non-Events (57)	Events (68)	*P* value
Age (year)	38.00 (30.00–51.00)	36.00 (30.00–47.00)	40.50 (29.00–54.50)	0.295
Female (%)	113 (90.4)	53 (93.0)	60 (88.2)	0.370
Height (cm)	160.00 (155.00–164.00)	160.00 (155.00–164.00)	159.50 (155.00–163.00)	0.739
Weight (kg)	55.00 (48.00–63.00)	54.00 (47.00–65.00)	55.00 (48.00–60.00)	0.868
BMI (kg/m^2^)	21.50 (19.56–23.34)	21.36 (19.28–23.88)	21.63 (19.79–23.16)	0.998
6MWD (m)	389.23 (311.60–465.80)	426.40 (354.49–495.00)	366.77 (288.16–432.42)	0.010
Subgroups of PH				0.102
IPAH	27 (21.6)	8 (14.0)	19 (27.9)	
CTD-PAH	65 (52.0)	32 (56.1)	33 (48.5)	
CHD-PAH	17 (13.6)	8 (14.0)	9 (13.2)	
PoPH	5 (4.0)	1 (1.8)	4 (5.9)	
CTEPH	11 (8.8)	8 (14.0)	3 (4.4)	
WHO-FC (%)				0.016
I/II	85 (68.0)	45 (78.9)	40 (58.8)	
III/IV	40 (32.0)	12 (21.1)	28 (41.2)	
Initial therapy (%)				0.551
Monotherapy	78 (62.9)	37 (64.9)	41 (61.2)	
Dual Therapy	40 (32.3)	18 (31.6)	22 (32.8)	
Triple Therapy	6 (4.8)	2 (3.5)	4 (6.0)	
Right Heart Catheterization				
RAP (mm Hg)	9.00 (6.00–12.00)	9.00 (6.00–13.00)	9.00 (6.00–12.00)	0.684
mPAP (mmHg)	50.00 (43.00–60.00)	51.00 (46.00–62.00)	49.50 (41.00–58.00)	0.095
PAWP (mmHg)	12.00 (9.00–14.00)	13.00 (10.00–14.00)	12.00 (8.00–13.00)	0.061
CO (L/min)	4.12 (3.20–5.00)	4.30 (3.10–4.90)	4.02 (3.30–5.10)	0.858
Cardiac index (L/min/m^2^)	2.60 (2.00–3.30)	2.60 (2.00–3.30)	2.54 (2.02–3.42)	0.911
PVR (Wood units)	10.00 (6.27–15.00)	10.00 (7.09–15.11)	9.89 (5.51–14.04)	0.457
SvO_2_ (%)	68.00 (61.00–74.00)	68.00 (62.00–73.00)	70.00 (61.00–74.00)	0.497
Echocardiography				
RV Diameter (mm)	45.00 (41.00–48.00)	46.00 (41.00–48.00)	44.50 (40.75–49.25)	0.565
TAPSE (mm)	16.00 (14.00–18.39)	16.24 (14.00–18.39)	16.00 (14.06–18.42)	0.903
PASP (mmHg)	78.00 (62.00–93.00)	76.00 (63.00–93.00)	78.10 (58.50–93.50)	0.777
TAPSE/PASP	0.21 (0.16–0.29)	0.20 (0.16–0.29)	0.21 (0.16–0.29)	0.659
Laboratory parameters				
TC (mmol/L)	4.09 (3.34–4.70)	4.20 (3.62–4.86)	3.80 (3.09–4.45)	0.024
TG (mmol/L)	1.33 (1.02–1.80)	1.40 (1.03–1.82)	1.29 (1.02–1.77)	0.695
HDL-C (mmol/L)	2.44 (1.84–2.88)	2.52 (2.07–3.01)	2.41 (1.83–2.83)	0.323
LDL-C (mmol/L)	1.02 (0.79–1.32)	1.16 (0.93–1.45)	0.90 (0.68–1.17)	<0.001
CRP (mg/L)	3.45 (0.99–6.94)	2.72 (0.70–5.36)	4.73 (2.50–8.00)	0.006
WBC (10^9^/L)	6.30 (4.82–8.07)	6.29 (4.66–8.10)	6.36 (4.87–7.86)	0.645
BNP (pg/mL)	200.00 (80.00–455.00)	218.00 (82.40–514.00)	182.00 (79.95–434.75)	0.723
AIP	0.15 (−0.08–0.32)	0.11 (−0.13–0.27)	0.20 (−0.01–0.34)	0.043

Data are shown as n (%), or median (interquartile range). BMI, body mass index; 6MWD, 6-min walk distance; PH, pulmonary hypertension; PAH, pulmonary arterial hypertension; IPAH, idiopathic PAH; CTD-PAH, connective tissue disease-associated PAH; CHD-PAH, congenital heart disease-associated PAH; PoPH, portopulmonary hypertension; CTEPH, chronic thromboembolic PH. WHO-FC, WHO cardiac functional class; RAP, right atrial pressure; mPAP, mean pulmonary artery pressure; PAWP, pulmonary artery wedge pressure; CO, cardiac output; PVR, pulmonary vascular resistance; SvO_2,_ mixed venous oxygen saturation; RV, right ventricle; TAPSE, tricuspid annular plane systolic excursion; PASP, pulmonary artery systolic pressure; TC, total cholesterol; TG, triglyceride; HDL-C, high-density lipoprotein cholesterol; LDL-C, low-density lipoprotein cholesterol; CRP, C-reactive protein; WBC, white blood cell; BNP, brain natriuretic peptide; AIP, atherogenic index of plasma.

### Association between AIP and prognosis in PH patients

**Figure 1:**
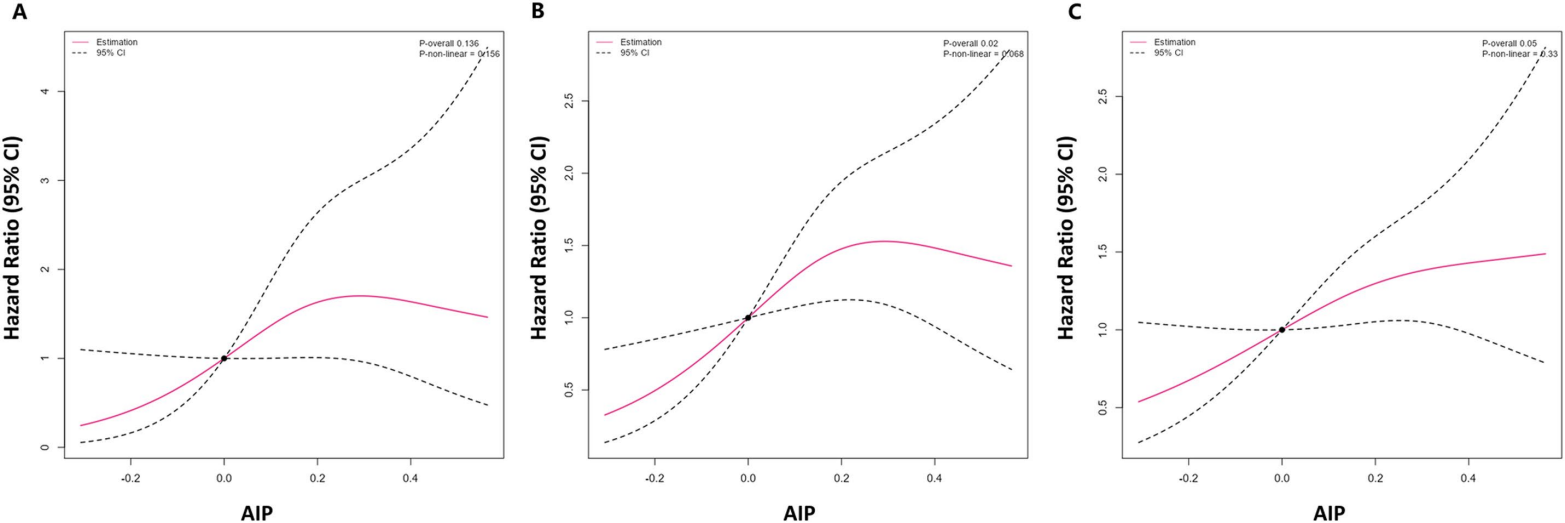
Restricted cubic spline analysis of AIP and 1-year **(A)**, 3-year **(B)**, and 5-year **(C)** risk of clinical deterioration events in PH patients. AIP, atherogenic index of plasma; PAH, pulmonary hypertension.

To explore the linear relationship between AIP and the risk of clinical worsening events in PH patients, restricted cubic spline analysis was performed (Figure 1). As shown in Figure 1, there was an inverted U-shaped correlation between AIP and the 1-year and 3-year risk of clinical worsening events in PH patients, with an initial positive correlation followed by a negative correlation. However, there was a significant positive linear correlation between AIP and the 5-year clinical outcome in PH patients.

The optimal cutoff value of AIP for predicting the risk of clinical worsening events in PH patients was estimated using the optimal *p*-value method, which was 0.144. Cox regression analysis was performed on AIP as a continuous variable and as a categorical variable in relation to the clinical outcome of PH patients (Table 2). As a continuous variable, AIP was not significantly associated with the risk of clinical worsening events in PH patients at 1-year follow-up, but reversed at 3-year and 5-year follow-up. In the crude model, continuous AIP was significantly associated with the risk of clinical worsening events, with hazard ratios (HRs) and 95% confidential intervals (CIs) of 3.399 (1.333–8.664) at 3-year follow-up and 2.829 (1.222–6.548) at 5-year follow-up. After adjusting for age and gender, the above results remained significant, with no change in the HRs and 95% CIs. After further adjusting for BMI, WHO-FC, PH initial therapy, BNP, TC, LDL-C, CRP, WBC, RAP, PAWP, PVR, SvO2, and TAPSE/PASP, the HRs and 95% CIs for continuous AIP were 3.069 (1.180–7.982) at 3-year and 2.426 (1.021–5.763) at 5-year follow-up.

**Table 2:** Multivariate cox regression of AIP for clinical deterioration events in PH patients.

Variables	Hazard ratio (95% confidence interval)					
	Model 1	*P* value	Model 2	*P* value	Model 3	*P* value
1-year						
AIP, continuous	3.851 (0.914–16.227)	0.066	3.851 (0.914–16.227)	0.066	3.793 (0.742–19.394)	0.109
AIP, category	3.309 (1.321–8.290)	0.011	3.309 (1.321–8.290)	0.011	2.805 (1.057–7.444)	0.038
3-year						
AIP, continuous	3.399 (1.333–8.664)	0.010	3.399 (1.333–8.664)	0.010	3.069 (1.180–7.982)	0.021
AIP, category	2.383 (1.368–4.151)	0.002	2.383 (1.368–4.151)	0.002	2.166 (1.236–3.795)	0.007
5-year						
AIP, continuous	2.829 (1.222–6.548)	0.015	2.829 (1.222–6.548)	0.015	2.426 (1.021–5.763)	0.045
AIP, category	2.124 (1.298–3.476)	0.003	2.124 (1.298–3.476)	0.003	1.834 (1.110–3.032)	0.018

Model 1: no adjustment; Model 2: adjusted for age and sex. Model 3: adjusted for model 2 plus BMI, WHO-FC, initial therapy, BNP, TC, LDL-C, CRP, WBC, RAP, PAWP, PVR, SvO_2_, and TAPSE/PASP. AIP, atherogenic index of plasma; PH, pulmonary hypertension.

As a categorical variable, AIP was significantly associated with the risk of clinical worsening events in PH patients at 1-year, 3-year, and 5-year follow-up. After adjusting for age and gender, the HRs and 95% CIs for categorical AIP were 3.309 (1.321–8.290) at 1-year, 2.383 (1.368–4.151) at 3-year, and 2.124 (1.298–3.476) at 5-year follow-up. After full adjustment of variables, the HRs and 95% CIs for categorical AIP were 2.805 (1.057–7.444) at 1-year, 2.166 (1.236–3.795) at 3-year, and 1.834 (1.110–3.032) at 5-year follow-up.

**Figure 2:**
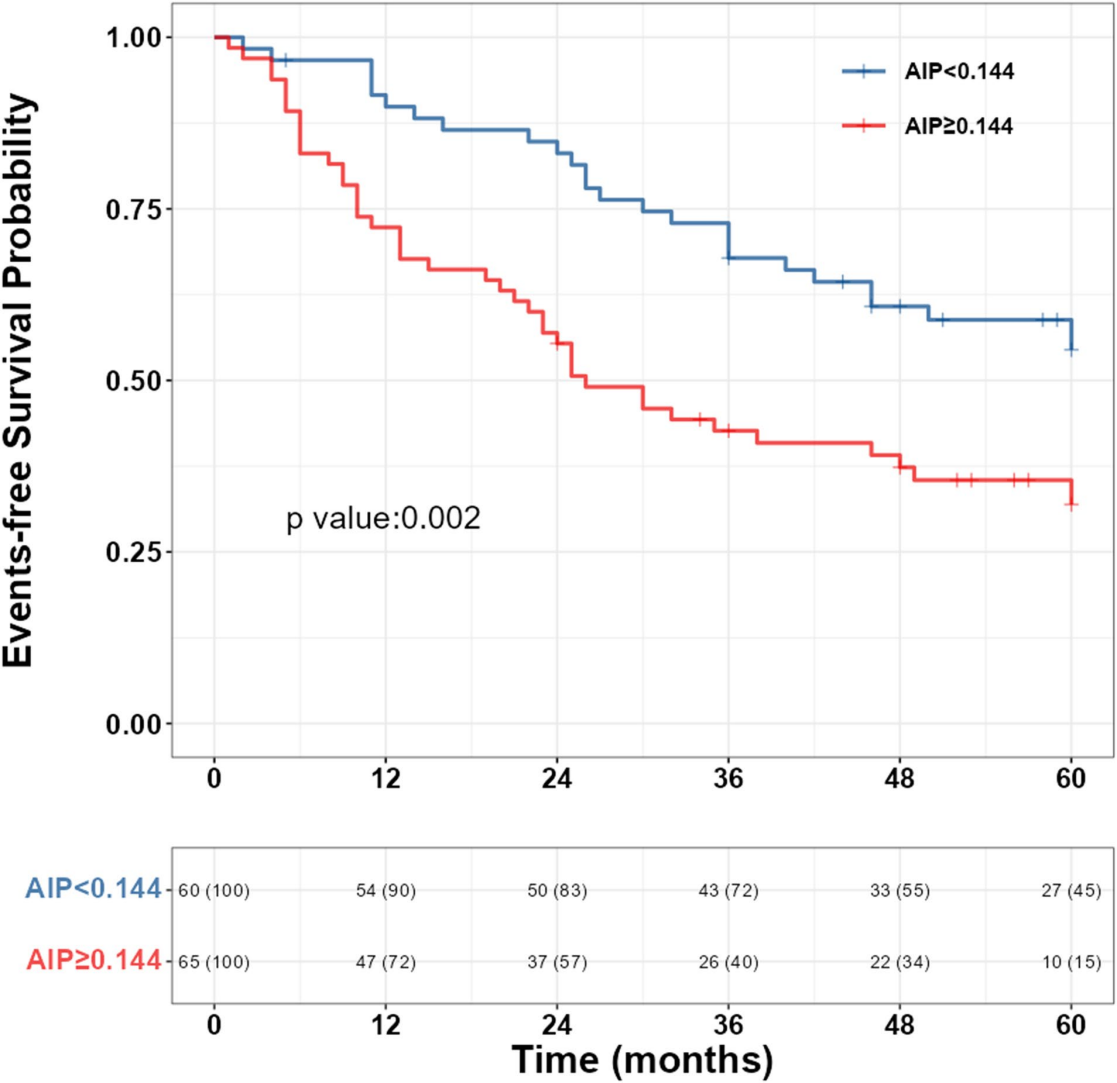
Kaplan–Meier survival curve of AIP and the risk of clinical deterioration events in PH patients. AIP, atherogenic index of plasma; PAH, pulmonary hypertension.

The 1-year, 3-year, and 5-year incidence rates of clinical worsening events in PH patients in this study were 20.0, 44.8, and 54.4%, respectively. Kaplan–Meier survival curves were used to compare the survival rate of AIP for the risk of clinical worsening events in PH patients (Figure 2), and the results showed a significantly increased risk of clinical worsening events in the high AIP group (*p* = 0.002).

### Construction and validation of nomogram

**Figure 3:**
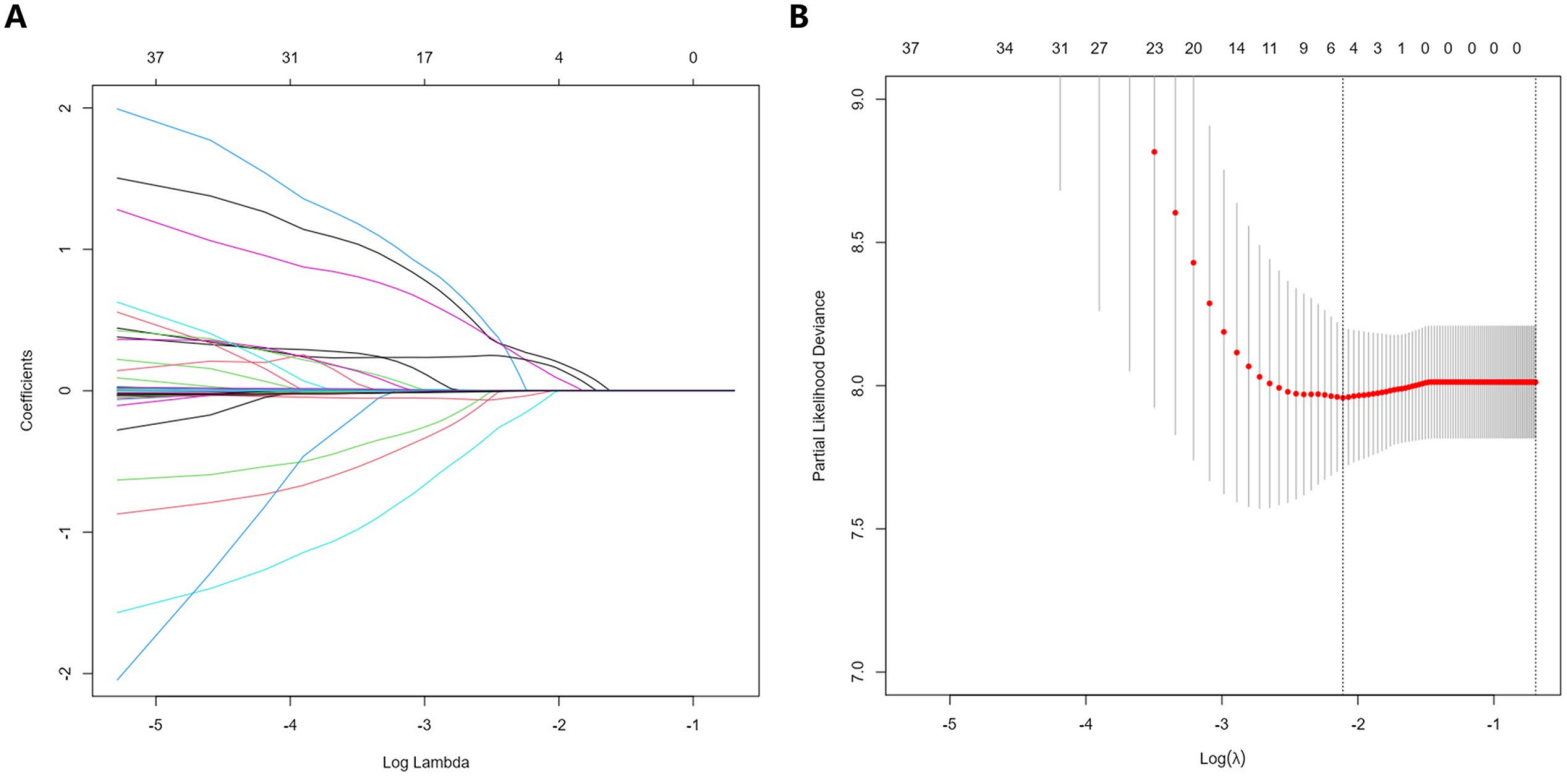
Selection of potential predictors of LASSO regression in PH patients. **(A)** The coefficient shrinkage process for various variables. **(B)** A cross-validation process to ascertain the optimal penalty parameter λ. LASSO, least absolute shrinkage and selection operator; PH, pulmonary hypertension.

**Figure 4:**
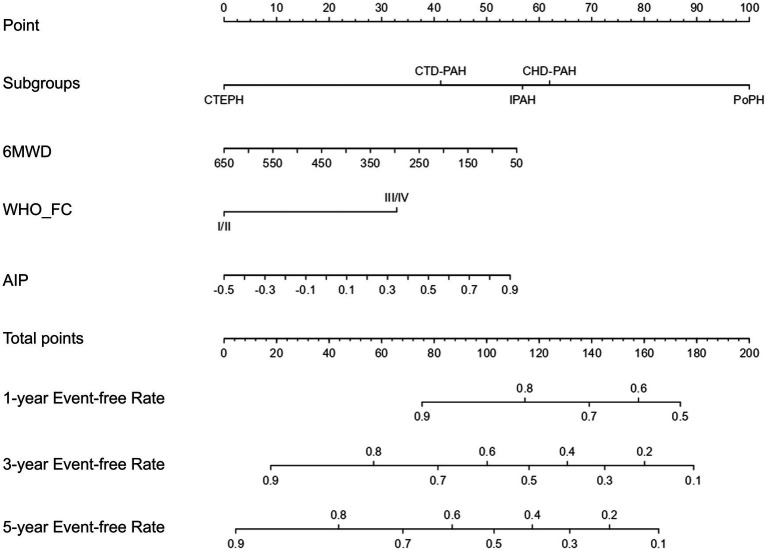
The nomogram for clinical deterioration prediction in PH patients. Subgroups, PH subtypes; 6MWD, 6-min walk distance; WHO_FC, WHO cardiac functional class; AIP, atherogenic index of plasma; PH, pulmonary hypertension.

LASSO regression was used to select potential variables, with ten-fold cross-validation and the penalty factor lambda selected as lambda.min. When the best lambda.min was 0.121, four prognostic variables (AIP, PH subgroups, 6MWD, and WHO-FC) were selected (Figure 3). Based on these variables, a nomogram was constructed for predicting clinical worsening events in PH patients (Figure 4). The results of the nomogram showed that CTEPH scored 0, connective tissue disease-associated PAH scored 41, IPAH scored 57, congenital heart disease-associated PAH scored 62, and PoPH scored 100. As the 6MWD increased, the score decreased gradually from 56 to 0. The score was 0 for WHO functional class I/II and 33 for class III/IV. With increasing AIP, the score increased gradually from 0 to 54.

**Figure 5:**
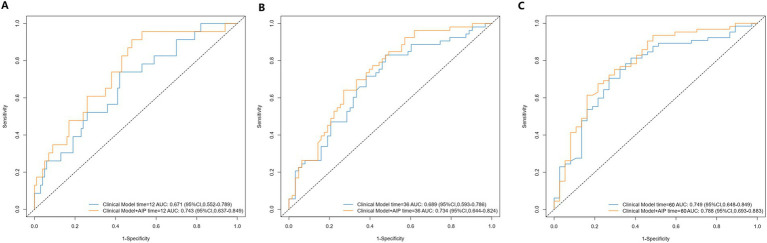
Receiver operating characteristic curves of AIP for 1-year **(A)**, 3-year **(B)**, and 5-year **(C)** adverse outcomes.AIP, atherogenic index of plasma. Clinical model includes variables of PH subtypes, 6-min walk distance, and WHO cardiac functional class.

To assess the predictive ability of the established model, we plotted the ROC curves for the clinical index model (including PH subtype, WHO functional class, and 6MWD) and the comprehensive model (clinical index model + AIP) for predicting clinical worsening events in PH patients (Figure 5). The 1-year, 3-year, and 5-year areas under the ROC curve (AUC) for the clinical index model alone were 0.671 (0.552–0.789), 0.689 (0.593–0.786), and 0.749 (0.648–0.849), respectively. The 1-year, 3-year, and 5-year AUC for the comprehensive model were 0.743 (0.637–0.849), 0.734 (0.644–0.824), and 0.788 (0.693–0.883), respectively. Although the *p*-values were not significant (all *p* > 0.05), the AUC values for 1-year, 3-year, and 5-year all increased. Due to the insensitivity of the ROC curve to the addition of new indices, we further introduced the NRI and IDI to evaluate the changes in the predictive ability of the clinical index model with the addition of AIP for clinical worsening events (Table 3). The NRI for 1-year, 3-year, and 5-year after introducing the model was 0.318 (0.027–0.498) (*p* = 0.040), 0.306 (0.039–0.459) (*p* < 0.001), and 0.337 (0.001–0.502) (*p* = 0.040), respectively, and the IDI was 0.058 (0.001–0.129) (*p* < 0.001), 0.049 (0.006–0.097) (*p* = 0.020), and 0.053 (−0.005–0.117) (*p* = 0.099), respectively. The NRI and IDI results showed a significant improvement in the predictive ability of the clinical index model after the introduction of AIP.

**Table 3:** Receiver operating characteristic and reclassification analyses for AIP to discriminate the risk stratification of pulmonary hypertension.

Model	AUC (95% CI)	P for comparison	NRI	*P* value	IDI	*P* value
1-year						
Clinical model*	0.671 (0.552–0.789)	Reference	Reference	Reference	Reference	Reference
Clinical model +AIP	0.743 (0.637–0.849)	0.129	0.318 (0.027–0.498)	0.040	0.058 (0.001–0.129)	<0.001
3-year						
Clinical model*	0.689 (0.593–0.786)	Reference	Reference	Reference	Reference	Reference
Clinical model +AIP	0.734 (0.644–0.824)	0.371	0.306 (0.039–0.459)	<0.001	0.049 (0.006–0.097)	0.020
5-year						
Clinical model*	0.749 (0.648–0.849)	Reference	Reference	Reference	Reference	Reference
Clinical model +AIP	0.788 (0.693–0.883)	0.484	0.337 (0.001–0.502)	0.040	0.053 (−0.005–0.117)	0.099

^*^Clinical model: including variables of PAH subtypes, WHO cardiac functional class, and 6-min walk distance. AIP, atherogenic index of plasma; AUC, areas under the ROC curve; NRI, net reclassification improvement; IDI, integrated discrimination improvement.

## Discussion

In this retrospective analysis, our study for the first time explored the association of AIP with the risk of clinical worsening events in PH patients. The findings showed a significant correlation between elevated AIP levels and increased risk of clinical deterioration, which is linearly increasing in the long-term. The nomogram based on AIP showed good prognostic performance for PH patients. Our results suggest that AIP could serve as a valuable prognostic biomarker in PH patients, potentially guiding therapeutic strategies and improving patient management.

The mechanism underlying the correlation of AIP with clinical worsening in PH is unclear, but it may be related to both inflammation and atherosclerosis. Inflammation plays an important role in the pathogenesis and prognostic survival of patients with PH (16, 17). Accumulating evidence suggests that chronic inflammation could accelerate the proliferation of pulmonary vascular endothelial cells and smooth muscle cells and lead to pulmonary artery constriction, which in turn promotes the occurrence and development of PAH (18). AIP is not only a representative of blood lipids in atherosclerosis, but also a brand-new indicator of systemic inflammation, because inflammatory cytokines could increase TG and reduce HDL-C levels (7, 19). Inflammatory cytokines could increase TG concentrations by increasing fatty acid synthesis and accelerating lipolysis, while modifying TG lipase could reduce HDL-C levels (19–21). Additionally, animal experiments and clinical studies have shown that insulin resistance is a risk factor and a prognostic biomarker for PH (22, 23). Taken together, AIP may serve not only as a marker of systemic inflammation and atherosclerosis but also as a mediator in PH development, warranting further investigation into these dual roles.

Lipid-lowering therapy may be beneficial for long-term prognostic survival in PH patients. Early animal experiments have shown that statins could alleviate or even reverse the progression of pulmonary hypertension (24–26). The underlying therapeutic mechanism of statins may be achieved by inhibiting the proliferation of smooth muscle cells and promoting their apoptosis (26). However, clinical studies have shown contrasting results and statins have not significantly improved clinical outcomes in PH patients (27–29). Previous clinical studies on statins have only 6 months of short-term outcomes, but our study displays that the 1-year and 3-year prognostic risk of PH patients increases first and then decreases as AIP increases, while the 5-year prognostic risk increases monotonously, suggesting the long-term benefits of lipid-lowering therapy. Furthermore, the main role of statins is to lower cholesterol, especially LDL-C, which is not in conflict with our results that lowering TG and improving HDL-C have a good prognosis effect on PH. Our findings may open up new ideas for lipid-lowering therapy in the management of PH patients. Future clinical studies should further explore whether different types of lipid-lowering treatments could improve the long-term prognosis of PH patients.

Our findings have important clinical implications for the management and prognosis of PH patients. Previous studies have focused on traditional risk factors and biomarkers of PH prognosis, such as BNP levels, 6MWD, and WHO-FC (30–32). Our study investigated the potential relationship of AIP, as a marker of inflammation and atherosclerosis, with the prognosis of PH patients, while also analyzing its role in routine clinical evaluations. The nomogram AUC showed no significant improvement after the addition of AIP to the clinical model, which may be attributed to the sample size limitation of this study and the insensitivity of the ROC curve to absolute risk changes (33, 34). To more accurately assess the role of AIP, we utilized two new metrics, namely NRI and IDI, to evaluate the incremental value of introducing AIP into the clinical model (35). The results of NRI and IDI showed a significant enhancement in the predictive ability of the model after adding AIP. Although the relationship between AIP and the prognosis of PH is affected by many confounding factors, we believe that our results are relatively robust after full adjustment of the variables (demographic information, blood parameters, RHC parameters, and ultrasound indicators). Prior investigators have highlighted the importance of lipid metabolism in cardiovascular disease (36–38), and our findings extend this understanding to PH, suggesting that lipid profile management may be an integral part of PH treatment regimens. Considering the complexity and multifactorial nature of PH (39), integrating AIP into existing prognostic models may improve the utility in terms of clinical decision-making accuracy.

### Limitation

One of the primary limitations of this study is the relatively small sample size of 125 patients, which may limit the generalizability of our findings to the broader population of PH patients. Additionally, the study was conducted at a single center, which may introduce bias related to local patient demographics and treatment protocols. Another limitation is the retrospective design, which inherently carries risks of missing or incomplete data, as well as potential recall bias. Due to the complexity of clinical conditions, the use of anti-inflammatory or immunosuppressive drug during follow-up may introduce some bias into the relationship between AIP and PH prognosis. While the study included a comprehensive collection of clinical and laboratory data, there may be unmeasured confounders that could influence the association between AIP and clinical outcomes in PH patients. Lastly, the conclusion of this study was based on the PH diagnostic standard of 25 mmHg, and whether it could be applicable to the diagnostic standard of 20 mmHg in the latest 2022 PH guidelines (40) remains to be investigated in future research. Future studies should aim to include larger, multi-center cohorts to validate these findings and enhance their applicability to diverse patient populations.

## Conclusion

In conclusion, our research provides novel insights into the prognostic value of AIP in PH, paving the way for more comprehensive management strategies that include lipid profile optimization. The nomogram including PH subgroups, 6MWD, WHO-FC, and AIP showed good prognostic performance for estimating adverse clinical events in PH patients. Future investigations should aim to expand on these findings and explore the therapeutic potential of modulating lipid metabolism in improving the prognosis of PH patients.

## Data Availability

The raw data supporting the conclusions of this article will be made available by the authors, without undue reservation.

## References

[1] Tello K Seeger W Naeije R Vanderpool R Ghofrani HA Richter M. Right heart failure in pulmonary hypertension: diagnosis and new perspectives on vascular and direct right ventricular treatment. Br J Pharmacol. (2021) 178:90–107. doi: 10.1111/bph.14866, PMID: 31517994

[2] Hoeper MM Humbert M Souza R Idrees M Kawut SM Sliwa-Hahnle K. A global view of pulmonary hypertension. Lancet Respir Med. (2016) 4:306–22. doi: 10.1016/s2213-2600(15)00543-3, PMID: 26975810

[3] Benza RL Miller DP Barst RJ Badesch DB Frost AE McGoon MD. An evaluation of long-term survival from time of diagnosis in pulmonary arterial hypertension from the REVEAL registry. Chest. (2012) 142:448–56. doi: 10.1378/chest.11-1460, PMID: 22281797

[4] Dobiásová M Frohlich J. The plasma parameter log (TG/HDL-C) as an atherogenic index: correlation with lipoprotein particle size and esterification rate in apoB-lipoprotein-depleted plasma (FER (HDL)). Clin Biochem. (2001) 34:583–8. doi: 10.1016/s0009-9120(01)00263-6, PMID: 11738396

[5] Tybjærg-Hansen A Nordestgaard BG Christoffersen M. Triglyceride-rich remnant lipoproteins are more atherogenic than LDL per particle: is this important? Eur Heart J. (2023) 44:4196–8. doi: 10.1093/eurheartj/ehad419, PMID: 37403539

[6] Annema W von Eckardstein A. High-density lipoproteins. Multifunctional but vulnerable protections from atherosclerosis. Circ J. (2013) 77:2432–48. doi: 10.1253/circj.cj-13-1025, PMID: 24067275

[7] Jonas K Magoń W Podolec P Kopeć G. Triglyceride-to-high-density lipoprotein cholesterol ratio and systemic inflammation in patients with idiopathic pulmonary arterial hypertension. Med Sci Monit. (2019) 25:746–53. doi: 10.12659/msm.912766, PMID: 30683836 PMC6359883

[8] Alifu J Xiang L Zhang W Qi P Chen H Liu L. Association between the atherogenic index of plasma and adverse long-term prognosis in patients diagnosed with chronic coronary syndrome. Cardiovasc Diabetol. (2023) 22:255. doi: 10.1186/s12933-023-01989-z, PMID: 37735427 PMC10515024

[9] Huang H Yu X Li L Shi G Li F Xiao J. Atherogenic index of plasma is related to coronary atherosclerotic disease in elderly individuals: a cross-sectional study. Lipids Health Dis. (2021) 20:68. doi: 10.1186/s12944-021-01496-8, PMID: 34247637 PMC8273949

[10] Rabiee Rad M Ghasempour Dabaghi G Darouei B Amani-Beni R. The association of atherogenic index of plasma with cardiovascular outcomes in patients with coronary artery disease: a systematic review and meta-analysis. Cardiovasc Diabetol. (2024) 23:119. doi: 10.1186/s12933-024-02198-y, PMID: 38566139 PMC10986012

[11] Shahdadian F Saneei P Lotfi K Feizi A Askari G Safavi SM. Association of plant-based diets with adropin, atherogenic index of plasma, and metabolic syndrome and its components: a cross-sectional study on adults. Front Nutr. (2023) 10:1077709. doi: 10.3389/fnut.2023.1077709, PMID: 37113295 PMC10128915

[12] Zhang Y Chen S Tian X Xu Q Xia X Zhang X. Elevated atherogenic index of plasma associated with stroke risk in general Chinese. Endocrine. (2024) 84:934–42. doi: 10.1007/s12020-023-03677-0, PMID: 38197990

[13] Sun M Liang C Lin H Chen Z Wang M Fang S. Association between the atherogenic index of plasma and left ventricular hypertrophy in patients with obstructive sleep apnea: a retrospective cross-sectional study. Lipids Health Dis. (2024) 23:185. doi: 10.1186/s12944-024-02170-5, PMID: 38867215 PMC11167813

[14] Yin B Wu Z Xia Y Xiao S Chen L Li Y. Non-linear association of atherogenic index of plasma with insulin resistance and type 2 diabetes: a cross-sectional study. Cardiovasc Diabetol. (2023) 22:157. doi: 10.1186/s12933-023-01886-5, PMID: 37386500 PMC10311747

[15] Jonas K Waligóra M Magoń W Zdrojewski T Stokwiszewski J Płazak W. Prognostic role of traditional cardiovascular risk factors in patients with idiopathic pulmonary arterial hypertension. Arch Med Sci. (2019) 15:1397–406. doi: 10.5114/aoms.2018.79242, PMID: 31749867 PMC6855165

[16] Huertas A Tu L Humbert M Guignabert C. Chronic inflammation within the vascular wall in pulmonary arterial hypertension: more than a spectator. Cardiovasc Res. (2020) 116:885–93. doi: 10.1093/cvr/cvz308, PMID: 31813986

[17] Yoo HHB Marin FL. Treating inflammation associated with pulmonary hypertension: an overview of the literature. Int J Gen Med. (2022) 15:1075–83. doi: 10.2147/ijgm.S295463, PMID: 35140509 PMC8820454

[18] Thoreau B Mouthon L. Pulmonary arterial hypertension associated with connective tissue diseases (CTD-PAH): recent and advanced data. Autoimmun Rev. (2023) 23:103506. doi: 10.1016/j.autrev.2023.103506, PMID: 38135175

[19] Khovidhunkit W Kim MS Memon RA Shigenaga JK Moser AH Feingold KR. Effects of infection and inflammation on lipid and lipoprotein metabolism: mechanisms and consequences to the host. J Lipid Res. (2004) 45:1169–96. doi: 10.1194/jlr.R300019-JLR200, PMID: 15102878

[20] Bi X Ai H Wu Q Fan Q Ding F Hu C. Insulin resistance is associated with interleukin 1β (IL-1β) in non-diabetic Hemodialysis patients. Med Sci Monit. (2018) 24:897–902. doi: 10.12659/msm.906269, PMID: 29436520 PMC5819312

[21] Zuliani G Volpato S Blè A Bandinelli S Corsi AM Lauretani F. High interleukin-6 plasma levels are associated with low HDL-C levels in community-dwelling older adults: the InChianti study. Atherosclerosis. (2007) 192:384–90. doi: 10.1016/j.atherosclerosis.2006.05.024, PMID: 16787648 PMC2645783

[22] Zamanian RT Hansmann G Snook S Lilienfeld D Rappaport KM Reaven GM. Insulin resistance in pulmonary arterial hypertension. Eur Respir J. (2009) 33:318–24. doi: 10.1183/09031936.00000508, PMID: 19047320 PMC2785883

[23] Hansmann G Wagner RA Schellong S Perez VA Urashima T Wang L. Pulmonary arterial hypertension is linked to insulin resistance and reversed by peroxisome proliferator-activated receptor-gamma activation. Circulation. (2007) 115:1275–84. doi: 10.1161/circulationaha.106.663120, PMID: 17339547

[24] Nishimura T Faul JL Berry GJ Vaszar LT Qiu D Pearl RG. Simvastatin attenuates smooth muscle neointimal proliferation and pulmonary hypertension in rats. Am J Respir Crit Care Med. (2002) 166:1403–8. doi: 10.1164/rccm.200203-268OC, PMID: 12406854

[25] Rakotoniaina Z Guerard P Lirussi F Goirand F Rochette L Dumas M. The protective effect of HMG-CoA reductase inhibitors against monocrotaline-induced pulmonary hypertension in the rat might not be a class effect: comparison of pravastatin and atorvastatin. Naunyn Schmiedeberg's Arch Pharmacol. (2006) 374:195–206. doi: 10.1007/s00210-006-0112-z, PMID: 17102939

[26] Gao YF Zhu XD Shi DM Jing ZC Li L Ma D. The effects of atorvastatin on pulmonary arterial hypertension and expression of p 38, p 27, and jab 1 in rats. Int J Mol Med. (2010) 26:541–7. doi: 10.3892/ijmm_00000497, PMID: 20818494

[27] Kawut SM Bagiella E Lederer DJ Shimbo D Horn EM Roberts KE. Randomized clinical trial of aspirin and simvastatin for pulmonary arterial hypertension: ASA-STAT. Circulation. (2011) 123:2985–93. doi: 10.1161/circulationaha.110.015693, PMID: 21593252 PMC3427737

[28] Barreto AC Maeda NY Soares RP Cícero C Lopes AA. Rosuvastatin and vascular dysfunction markers in pulmonary arterial hypertension: a placebo-controlled study. Braz J Med Biol Res. (2008) 41:657–63. doi: 10.1590/s0100-879x2008000800003, PMID: 18797697

[29] Zeng WJ Xiong CM Zhao L Shan GL Liu ZH Xue F. Atorvastatin in pulmonary arterial hypertension (APATH) study. Eur Respir J. (2012) 40:67–74. doi: 10.1183/09031936.00149011, PMID: 22362846

[30] Williams MH Handler CE Akram R Smith CJ Das C Smee J. Role of N-terminal brain natriuretic peptide (N-Tpro BNP) in scleroderma-associated pulmonary arterial hypertension. Eur Heart J. (2006) 27:1485–94. doi: 10.1093/eurheartj/ehi891, PMID: 16682379

[31] Reesink HJ van der Plas MN Verhey NE van Steenwijk RP Kloek JJ Bresser P. Six-minute walk distance as parameter of functional outcome after pulmonary endarterectomy for chronic thromboembolic pulmonary hypertension. J Thorac Cardiovasc Surg. (2007) 133:510–6. doi: 10.1016/j.jtcvs.2006.10.020, PMID: 17258590

[32] Savarese G Paolillo S Costanzo P D'Amore C Cecere M Losco T. Do changes of 6-minute walk distance predict clinical events in patients with pulmonary arterial hypertension? A meta-analysis of 22 randomized trials. J Am Coll Cardiol. (2012) 60:1192–201. doi: 10.1016/j.jacc.2012.01.083, PMID: 22995024

[33] Cook NR. Use and misuse of the receiver operating characteristic curve in risk prediction. Circulation. (2007) 115:928–35. doi: 10.1161/circulationaha.106.672402, PMID: 17309939

[34] Cook NR Ridker PM. Advances in measuring the effect of individual predictors of cardiovascular risk: the role of reclassification measures. Ann Intern Med. (2009) 150:795–802. doi: 10.7326/0003-4819-150-11-200906020-00007, PMID: 19487714 PMC2782591

[35] Pencina MJ D'Agostino RB Sr D'Agostino RB Vasan RS. Evaluating the added predictive ability of a new marker: from area under the ROC curve to reclassification and beyond. Stat Med. (2008) 27:157–72. doi: 10.1002/sim.2929, PMID: 17569110

[36] Brandts J Ray KK. Novel and future lipid-modulating therapies for the prevention of cardiovascular disease. Nat Rev Cardiol. (2023) 20:600–16. doi: 10.1038/s41569-023-00860-8, PMID: 37055535

[37] Deprince A Haas JT Staels B. Dysregulated lipid metabolism links NAFLD to cardiovascular disease. Mol Metab. (2020) 42:101092. doi: 10.1016/j.molmet.2020.101092, PMID: 33010471 PMC7600388

[38] Pirillo A Tokgözoğlu L Catapano AL. European lipid guidelines and cardiovascular risk estimation: current status and future challenges. Curr Atheroscler Rep. (2024) 26:133–7. doi: 10.1007/s11883-024-01194-7, PMID: 38430340

[39] Thenappan T Ormiston M Ryan J Archer S. Pulmonary arterial hypertension: pathogenesis and clinical management. BMJ. (2018) 360:j5492. doi: 10.1136/bmj.j5492, PMID: 29540357 PMC6889979

[40] Humbert M Kovacs G Hoeper MM Badagliacca R Berger RMF Brida M. 2022 ESC/ERS guidelines for the diagnosis and treatment of pulmonary hypertension. Eur Heart J. (2022) 43:3618–731. doi: 10.1093/eurheartj/ehac237, PMID: 36017548

